# The NEDD8 E3 ligase DCNL5 is phosphorylated by IKK alpha during Toll-like receptor activation

**DOI:** 10.1371/journal.pone.0199197

**Published:** 2018-06-29

**Authors:** Yann Thomas, Daniel C. Scott, Yosua Adi Kristariyanto, Jesse Rinehart, Kristopher Clark, Philip Cohen, Thimo Kurz

**Affiliations:** 1 Medical Research Council Protein Phosphorylation and Ubiquitylation Unit, School of Life Sciences, University of Dundee, Dundee, United Kingdom; 2 Department of Structural Biology, Howard Hughes Medical Institute, St. Jude Children’s Research Hospital, Memphis, Tennessee, United States of America; 3 Department of Cellular & Molecular Physiology, Yale University, New Haven, Connecticut, United States of America; 4 Systems Biology Institute, Yale University, West Haven, Connecticut, United States of America; University of South Florida College of Arts and Sciences, UNITED STATES

## Abstract

The activity of Cullin-RING ubiquitin E3 ligases (CRL) is regulated by NEDD8 modification. DCN-like proteins promote Cullin neddylation as scaffold-like E3s. One DCNL, DCNL5, is highly expressed in immune tissue. Here, we provide evidence that DCNL5 may be involved in innate immunity, as it is a direct substrate of the kinase IKKα during immune signalling. We find that upon activation of Toll-like receptors, DCNL5 gets rapidly and transiently phosphorylated on a specific N-terminal serine residue (S41). This phosphorylation event is specifically mediated by IKKα and not IKKβ. Our data for the first time provides evidence that DCNL proteins are post-translationally modified in an inducible manner. Our findings also provide the first example of a DCNL member as a kinase substrate in a signalling pathway, indicating that the activity of at least some DCNLs may be regulated.

## Introduction

Cullin–RING complexes (CRLs) are modular ubiquitin E3 enzymes that consist of a Cullin scaffold protein, which at its N-terminus interacts with substrate-specificity modules, and at its C-terminus binds to a small RING-finger protein (Rbx1 or Rbx2) that recruits the E2 enzyme [1]. Mammalian cells contain eight Cullin proteins, Cul1, Cul2, Cul3, Cul4A, Cul4B, Cul5, Cul7 and Cul9/Parc8 [2].

The assembly and activity of Cullin-based ligases is regulated through reversible conjugation of Nedd8, a ubiquitin-like protein, which is covalently attached to a conserved lysine residue in the Cullin backbone [3,4].

Like ubiquitination, neddylation of substrates is achieved by an enzymatic cascade involving the Nedd8-activating enzyme (NAE) APP-BP1 (ULA1)/UBA3 and two Nedd8-conjugating enzymes encoded by UBE2M and UBE2F. The Cullin-bound RING-finger protein (Rbx1/Rbx2) promotes auto-neddylation of the CRL complex, aided by DCN1-like proteins (defective in Cullin neddylation 1). DCNLs directly bind to the Cullin and the Nedd8 E2 enzyme to position them in a productive conformation for neddylation by Rbx1 [5] [6,7].

Human cells harbor 5 Dcn1-like proteins termed DCNL1–DCNL5 (also named DCUN1D 1–5 for defective in Cullin neddylation 1 domain-containing protein 1–5 or SCRRO1-5). These DCNLs have distinct amino-terminal domains, but share a conserved C-terminal potentiating neddylation (PONY) domain, which is necessary and sufficient for optimal Cullin neddylation *in vitro* and *in vivo* [8] [9]. The Cullin interaction surface at the C-terminus of the PONY domain, the DAD patch (D226, A253, D259 in *Saccharomyces cerevisiae* Dcn1), is conserved in all human DCNLs. Like the yeast Dcn1, DCNL1 and DCNL2 harbor a predicted amino-terminal UBA domain, which directly binds ubiquitin [6]. DCNL3 harbors a myristoylation site in its amino-terminal domain, which localizes the protein to the plasma membrane, where it recruits Cullin3 and stimulates its neddylation [10]. DCNL4 and DCNL5 contain a nuclear localization signal (NLS) in their amino terminal domain, which is in part responsible for their predominant nuclear localization [9].

In animals the expression of most DCNLs is restricted to certain tissues, indicating that they may play specific roles in defined physiological contexts, while in cultured cancer cells, all DCNLs seem to be expressed at similar levels [9].

Our previous work provided some leads to potentially explain the specificities between DCNL and Cullins, which could mainly be a result of their differential expression patterns [9]. In this study we provide another piece of evidence that is indicative of DCNL-specific regulatory mechanisms. We found that DCNL5 is mainly expressed in immune tissues and that during the innate immune response DCNL5 is transiently phosphorylated by IKKα on its N-terminal Serine 41. Although further work is required to uncover the role of this phosphorylation in immune signaling, the transient and regulated post translational modification of DCNL5 on a single residue in its amino terminal domain indicates that the phosphorylation of DCNL5 may have an important role in the regulation of innate immunity.

## Material and methods

### Materials

Protein kinase inhibitors were dissolved in DMSO and stored at -20°C as 10 mM solutions. The TLR agonists Pam3CSK4 and Poly (I:C) were purchased from Invivogen, lipopolysaccharide (LPS) from Alexis Biochemicals. Mouse IL1α was from Sigma-Aldrich.

### Antibodies

For immunoblotting we used mouse anti-Flag M2 (Sigma), anti-HA (Cell Signaling), anti-Actin (Millipore), anti-GAPDH (Cell Signaling), anti-Cullin1 (Invitrogen), anti-Cullin2 (Invitrogen), anti-DCNL1 (Sigma), anti- Iκbα (Cell signaling), anti-phosphoSer^933^ p105 (Cell signaling), anti-phosphoThr^172^ TBK1 (Cell signaling), anti-phosphoThr^180^/Tyr^182^ p38 (cell signaling), anti-phosphoThr^183^/Tyr^185^ JNKs (cell signaling), anti-phosphoTyr^701^ Stat1 (cell signaling), anti-phosphoSer^396^ IRF3 (cell signaling), anti-phosphoSer^180/181^ IKKα/β (Cell signaling), anti-p27 (cell signaling) and anti Histone H3 (cell signaling). Sheep polyclonal DCNL3 (S996C), DCNL4 (S997C) and DCNL5 antibodies (S424D) were raised against a bacterially expressed protein fragment corresponding to the specific amino-terminal domain of each protein fused to GST. The antibodies were purified from sheep serum by affinity chromatography on CH-Sepharose to which the peptide immunogen had been covalently coupled. Sheep polyclonal antibodies recognizing the phospho-epitope of DCNL5 (S441D), a C-terminal fragment of Cullin3 (S347D) and Cullin5 (S073D) and an amino terminal fragment of Cullin4a (S084D) were produced following the same protocol. These antibodies are available from "MRCPPU Reagents”, https://mrcppurreagents.dundee.ac.uk"

For immunofluorescence analyses, we used mouse anti-Flag M2 (Sigma) and a specific secondary antibody conjugated to Alexa Fluor 488 (1:1,000) from Molecular Probes.

### Cell Culture

U2OS, HEK293, MEFs and RAW264.7 were grown on DMEM (Dulbecco’s modified Eagle’s medium; GIBCO) supplemented with 10% FBS (fetal bovine serum; GIBCO) and 100 units/ml penicillin, 100 μg/ml streptomycin (Invitrogen).

Stably transfected Flp-In T-Rex-293 and U2Os cells were generated as [9]. The cells were stimulated with 1 μg/ml tetracycline overnight to induce expression of the desired fusion proteins.

### Cell extracts, immunoprecipitation and immunoblot analyses

Whole-cell extracts were prepared from mammalian cells by lysis in 50 mM Tris-HCl at pH7.4, 1 mM EDTA, 1 mM EGTA, 50 mM NaF, 5 mM sodium pyrophosphate, 10 mM sodium β-glycerol 1-phosphate, 1 mM sodium orthovanadate, 0.27 M sucrose, 1% Triton X-100, complete phosphatase inhibitor PhosSTOP (Roche) and 15 mM Iodoacetamide for 30 min on ice, before clarification by centrifugation. To detect protein in cell lysates, protein samples were separated by SDS-PAGE and transferred onto nitrocellulose membrane. Proteins were detected by immunoblotting and visualized by treating the blots with ECL (Millipore).

### RNA interference

siRNA transfection of U2Os was carried out using Lipofectamine^™^ RNAiMAX transfection Reagent. Conditions used for RNA transfection in these cells were as described previously [11] [12]. Cells were transfected with SMARTpool siRNA oligos against human proteins, the results were then confirmed with single siRNA oligos (Dharmacon).

RAW264.7 cells were transfected with 100 pmol of SMARTpool siRNA oligos against mouse DCNL5, DCNL1, DCNL4 or a non-targeting control (Dharmacon) by using AMAXA nucleofection. Cells were cultured for 24h before stimulation with 100ng/ml LPS. Cells were lysed for protein extraction or RNA extraction.

### qPCR

Total RNA was extracted from RAW264.7 macrophages using the MicroElute Total RNA kit (OMEGA Biotek/VWR) following the manufacturer’s instructions and quantitated by measuring the absorbance at 260 nm. 1 μg of total RNA was reversed transcribed into cDNA using the PrimeScript RT mastermix (Takara). cDNA (50 ng) was incubated with primers (100 nM) in a total volume of using the SsoFast EvaGreen Supermix (OMEGA Biotek) on a CFX384 real time system (Bio-Rad Laboratories). The amplified mRNA was measured using the DD Cycle Threshold (CT) method and the constitutively expressed 18S RNA as an internal control.

The primers used were the following:

DCUN1D5 F (Forward) TATTGCAGATCCCAGCCTCC
DCUN1D5 R (Reverse) TCTCCCAAGCAGCAGAGCTA
Iκbα F ACACGTGTCTGCACCTAG
Iκbα R TCAGACGCTGGCCTCCAAAC
18S F GTAACCCGTTGAACCCCATT
18S R CCATCCAATCGGTAGTAGCG
A20 F ACTGGAATGACGAATGG
A20 R CTTCTGAGGATGTTGCT
TNFα F CAGACCCTCACACTCAGATCATC
TNFα R GCTACAGGCTTGTCACTCG
IL10 F CCCTTTGCTATGGTGTCCTTTC
IL10 R GATCTCCCTGGTTTCTCTTCCC
IFNb F GGAAAAGCAAGAGGAAAGATTGAC
IFNb R CCACCATCCAGGCGTAGC
Isg15 F CAGGACGGTCTTACCCTTTCC
Isg15 R AGGCTCGCTGCAGTTCTGTAC
CXCL10 F CCTGCAGGATGATGGTCAAG

### *In vitro* phosphorylation reactions

*In vitro* phosphorylation reactions were performed in a volume of 25 μl containing 50 mM Tris-HCl pH 7.5, 0.1 mM EGTA, 1 mM DTT, 10 mM MgCl_2_, 0.1 mM g 32-ATP (Perkin Elmer), 20 μM of substrate and 0.83 μg IKKα or 0.29 μg IKKβ. The reaction was started by adding the kinase and was carried out for the times indicated at 30°C with constant agitation. The reaction was quenched by adding 10 μl sample buffer. GST-IKKα and GST-IKKβ were expressed in suspension HEK293FT cells, purified on GSH Sepharose and cleaved with preScission Protease (GE).

### Phospho-proteomics

RAW264.7 cells were labeled using the Stable Isotope Labelling of Amino Acids in Cell Culture (SILAC) method. Cells were treated with 2 μM MRT67307 or vehicle control for 1h and left unstimulated or stimulated with 1 μg/ml Pam3CSK4 for 30 min or 10 μg/ml Poly(I:C). The subsequent phospho-proteomic analyses were done as described previously [13].

### Co-translational phosphoserine incorporation into DCNL5

Strains were grown at 30 °C in LB media containing 0.08% glucose, 100 μg/ ml Ampicillin and 25 μg/ml Kanamycin to retain plasmids for Sep incorporation in EcAR7 bacterial strain as previously described [14]. Protein expression was induced by addition of 0.25 mM IPTG to the media and phosphoserine incorporation was facilitated by the addition of 2 mM Sep to the media. Cells were grown at 16°C for 20–24 hours and harvested by subsequent centrifugation. For protein purification, cells were suspended in lysis buffer (50 mM Tris-HCl pH 7.5, 500 mM NaCl, 0.5 mM EDTA, 0.5 mM EGTA, 10% glycerol, 5 mM DTT, 1mg/ml Lysozyme, 50 mM NaF, 1 mM Na_3_VO_4_, 25 U/ml benzonase and protease inhibitors (Roche)) for 30 minutes on ice. Cell extracts were obtained by centrifugation and applied to Glu-Seph affinity resin for 1 hour and washed several times with wash buffer (50 mM Tris-HCl pH 7.5, 500 mM NaCl, 0.5 mM EDTA, 0.5 mM EGTA, 10% glycerol, 5 mM DTT, 1mg/ml Lysozyme, 50 mM NaF, 1 mM Na_3_VO_4_). Proteins were eluted after an overnight incubation with preScission protease (GE) at 4°C.

The incorporation of the phospho-serine was verified by Mass fingerprinting (S6 Fig).

## Results

### DCNL5 is a substrate of IKKα during Toll like receptor (TLR) signaling

To gain further insight into a specific function of DCNLs, we focused on DCNL5, which is mainly expressed in Spleen, Thymus and Lymph Nodes of mice [9]. These organs are part of the immune system and contain several immune cell types such as T lymphocytes, B lymphocytes, dendritic cells and macrophages, which contribute to innate or adaptive immunity. The adaptive immune system is characterized by the ability to respond to pathogens that have not been previously encountered, to gear the response to the specific type of pathogen and to retain memory of the pathogenic encounter for subsequent challenge. This program is manifested by B and T lymphocytes of the immune system and the specially designed antigen receptors that are expressed by these cells [15]. In contrast to adaptive immunity, the innate immune system is the major contributor to acute inflammation induced by microbial infection or tissue damage. During infection by bacteria or viruses, components of these pathogen bind to TLRs (Toll-like receptors) on immune cells such as macrophages. This triggers the activation of signaling pathways that control the production of inflammatory mediators and interferons (IFNs) to combat the invading pathogen [16]. The production of inflammatory mediators in the innate immune system is triggered by activation of signaling pathways, including the NF-KB pathway. The canonical Iκb kinase (IKK) complex has many important roles, one of which is to control NF-KB signalling. It activates the NF-KB transcription factors and hence NF-KB-dependent transcription by phosphorylating the NF-KB inhibitor Iκbα. The phosphorylation of Iκbα licences it for ubiquitylation by the CRL SCF^βtrcp^ (Skp1/Cullin1/F-box b-transducin repeat containing protein) and its subsequent degradation by the proteasome [17]. NF-KB can then enter the nucleus and drive a pro-inflammatory transcription program.

**Fig 1:**
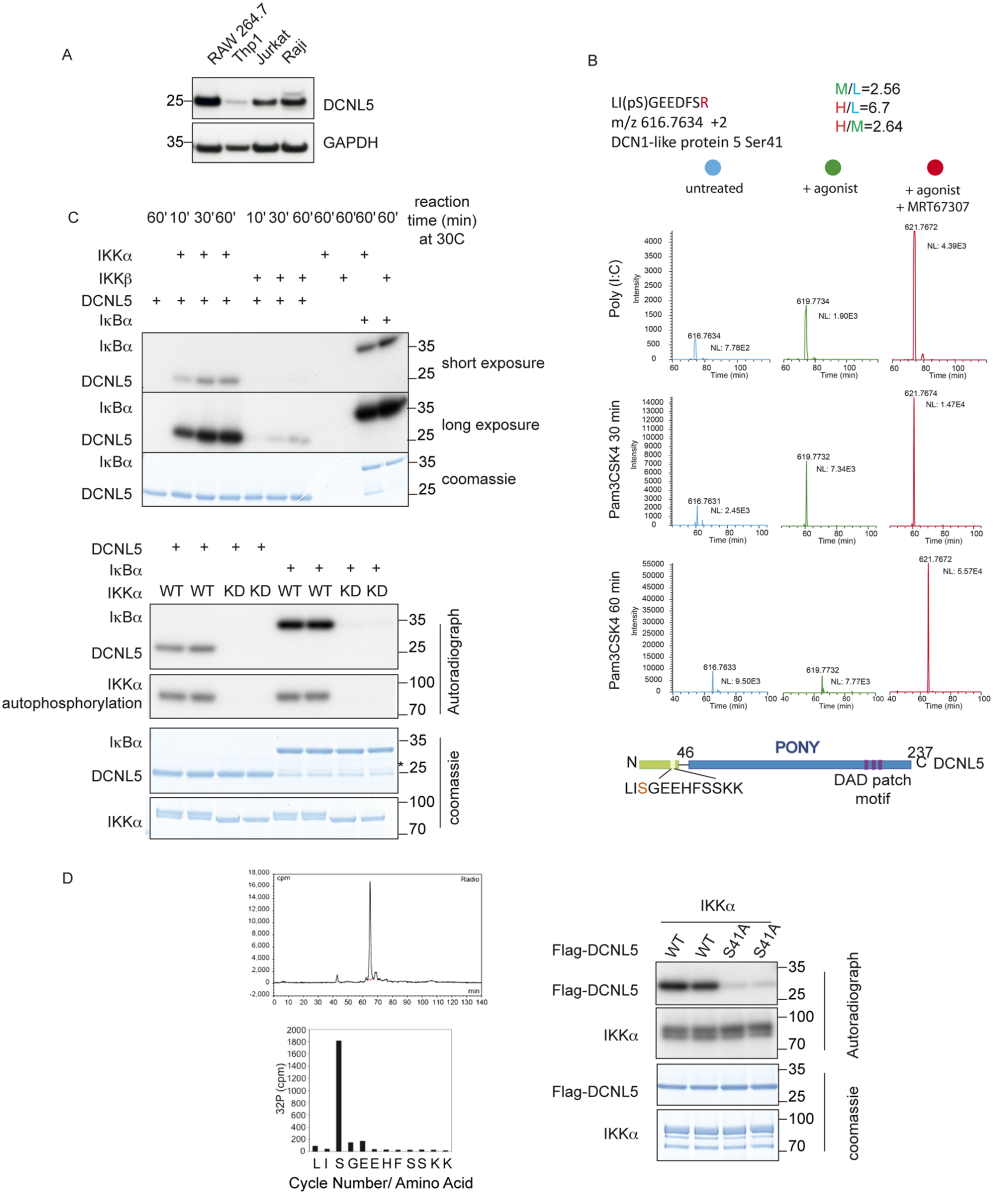
DCNL5 is phosphorylated on Serine 41 by IKKα. **(A**) Immunoblot analysis with the indicated antibodies of cell lysates from different cell lines. DCNL5 is strongly expressed in RAW264.7 (immortalized macrophages), Jurkat (immortalized T lymphocytes), and Raji (derived B lymphocytes) cells, while it is present at low levels in Thp1 cells (a monocyte-derived cell line). **(B**) Upper panel: Mass spectrum showing phosphopeptide precursor ions corresponding to DCNL5 (amino acids 39–48), which is phosphorylated at Ser41 in RAW264.7 macrophages treated with Poly (I:C) or Pam3CSK4, pre-treatment with MRT67307 (1 μM) for 1 hour enhance the phosphorylation. The results for unstimulated RAW264.7 macrophages are presented in blue (light), results for RAW264.7 macrophages stimulated with Poly (I:C) or Pam3CSK4 are in green (medium) and the results for RAW264.7 macrophages pre-treated with MRT67307 prior to stimulation with Poly (I:C) or Pam3CSK4 are depicted in red (heavy). For the Poly (I:C) treated samples, the ratio of labelled phosphopeptides in the different conditions quantified from MaxQUANT are given. Lower panel: Diagram of DCNL5 depicting the conserved C-ter PONY domain, its specific N-ter domain and the phosphorylated residue (red font; S41) uncovered by MS analysis. **(C)** Top panel: 1.4 μg of the purified recombinant DCNL5 was incubated with IKKα or IKKβ in the presence of **γ**32-ATP at 30°C for the times indicated. 1.4 μg of Iκbα was included as a positive control for IKKα and IKKβ. The stopped reactions were run on a gel and exposed to X-ray films. Right panel: same reactions as the top panel using IKKα WT or kinase dead mutant (S176A/S180A). The asterisk depicts degradation products from Iκbα that run at the same position as DCNL5. **(D)** *In vitro* phosphorylated DCNL5 was tryptic digested and separated by reverse chromatography followed by radioactivity measurement. Fractions corresponding to DCNL5 phosphopeptide were subjected for Edman sequencing. Flag tagged recombinant DCNL5 WT or S41A mutant was phosphorylated *in vitro* by IKKα WT in the presence of **γ**32-ATP at 30 °C for 30 minutes.

Due to its high levels in immune tissue, we reasoned that DCNL5 may be involved in immune signaling. We thus first determined the expression of DCNL5 in different immune tissue culture cell lines. Using Western Blot analysis we found that DCNL5 is strongly expressed in RAW264.7 (immortalized macrophages), Jurkat (immortalized T lymphocytes), and Raji (derived B lymphocytes) cells, while it seemed to be present at lower levels in Thp1 cells (a monocyte-derived cell line) (Fig 1A).

As protein kinases are the best-characterised regulators of immunity, we first determined if DCNL5 becomes phosphorylated during immune signaling. To this end, we performed a phospho-proteomic analysis of DCNL5 before and after induction of innate immune signaling pathways by stimulation with Poly (I:C) (Polyinosine-polycytidylic acid), a TLR3 agonist that signals via TRIF. Using SILAC labeled RAW264.7 macrophages we determined that DCNL5 shows increased phosphorylation on the amino-terminal Serine 41 residue after 30 minutes of stimulation (Fig 1B top panel). The same pattern of phosphorylation is observed using Pam3CSK4 (Pam3-Cys-Ser-Lys4), a TLR1/2 agonist that signals via Myd88 (Fig 1B middle panel). Thus, DCNL5 is phosphorylated in response to both TRIF and Myd88 dependent TLR pathway activation. After 60 minutes stimulation, the DCNL5 phosphorylation state returned to the unstimulated condition (Fig 1B bottom panel). Pre-treatment of cells with MRT67307 prior to stimulation further enhanced the level of phosphorylation and sustained phosphorylation beyond 60 minutes (Fig 1B right panels). MRT67307 specifically inhibits the IKKε/TBK1 kinases, which are involved in controlling the production of Type1 interferons but also participate in a negative regulatory loop that restricts the extent of activation of the IKK complex [18] [19]. This suggested that DCNL5 might be a substrate of the IKK complex (IKKα or IKKβ) in both TRIF-dependent and Myd88-dependent TLR pathways.

To test our hypothesis, we studied whether recombinant IKKα or IKKβ could phosphorylate recombinant DCNL5 *in vitro*. Only IKKα phosphorylated DCNL5 efficiently, while both kinases were able to phosphorylate their known substrate Iκbα equally well (Fig 1C, left panel). An inactive version of IKKα (S176/S180A) did not support phosphorylation of DCNL5, further demonstrating that IKKα activity is required for DCNL5 phosphorylation *in vitro* (Fig 1C, bottom panel) [20]. We next determined the phosphorylation site on DCNL5 after *in vitro* phosphorylation by phospho-mapping, reverse phase chromatography followed by Edman sequencing (Fig 1D, left panel) [21], which confirmed that IKKα phosphorylates DCNL5 on Serine 41 *in vitro*, the same site we previously identified by phospho-proteomics in cells. Consistent with S41 being the major phosphorylation site, mutation of this Serine to Alanine, to generate DCNL5 (S41A), abolished phosphorylation *in vitro* (Fig 1D, right panel).

**Fig 2:**
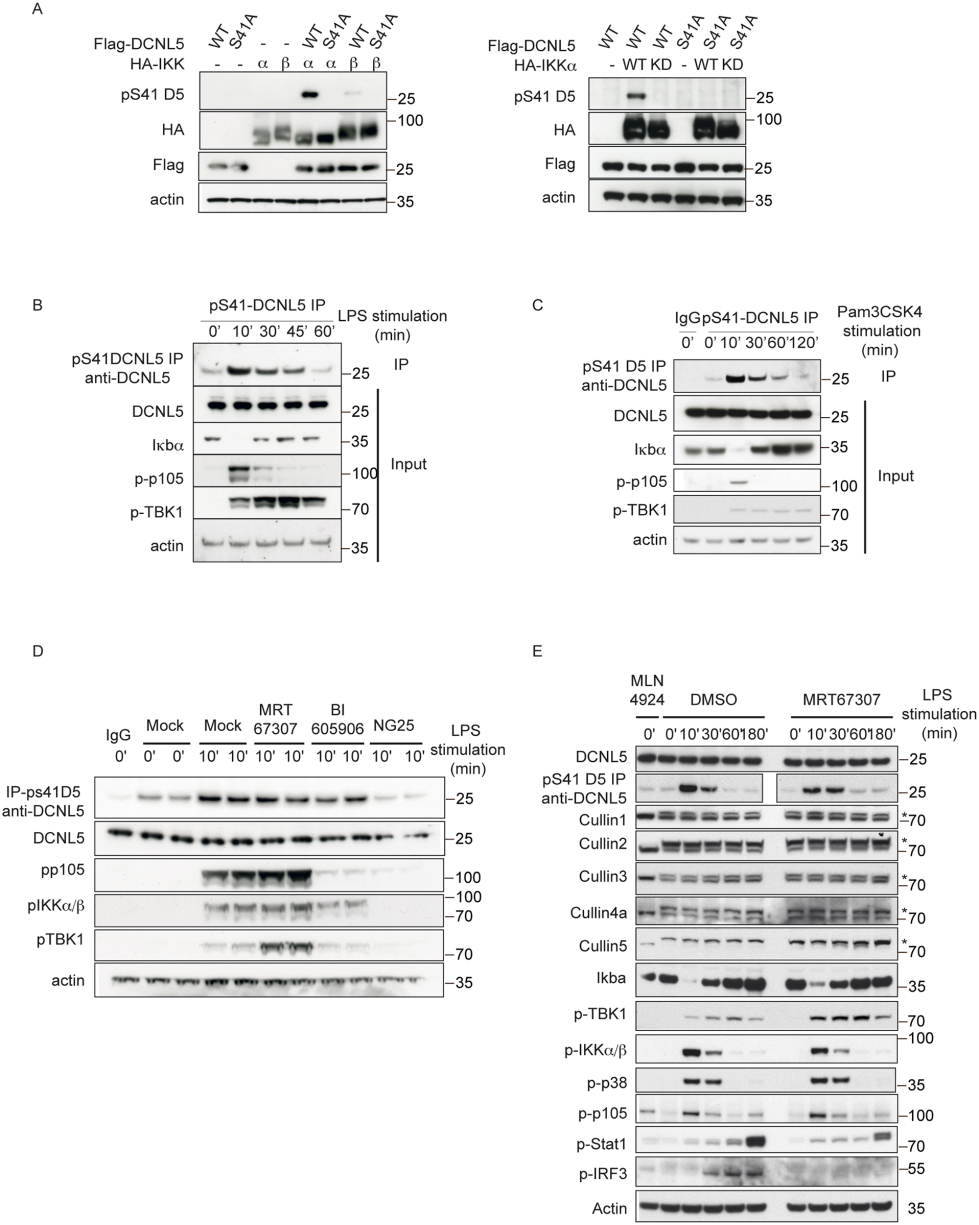
DCNL5 is phosphorylated on Serine 41 by IKKα in RAW264.7 macrophages upon TLR stimulation. (A) HEK293 cell lines were transfected with Flag tagged DCNL5 WT or S41A and HA-tagged IKKα/ β WT (left panel) or kinase dead mutant (right panel). The cell lysates (30 μg) were resolved on SDS-PAGE and analyzed by immunoblotting with indicated antibodies. **(B)** RAW264.7 macrophages were treated with 100 ng/ml LPS over the indicated time period and subjected to immunoblot analyses. To visualize the phosphorylated form of DCNL5, cell extracts were subjected to immunoprecipitation using the phospho-specific antibody and analyzed by immunoblot with a total DCNL5 antibody. **(C)** RAW264.7 macrophages were treated with 1 μg/ml Pam3CSK4 over the indicated time period and subjected to immunoblot analyses. To visualize the phosphorylated form of DCNL5, cell extracts were subjected to immunoprecipitation using the phospho-specific antibody and analyzed by immunoblot with a total DCNL5 antibody. **(D)** RAW264.7 macrophages were treated 1 hour with MRT67307 (1μM), BI605906 (10μM) or NG25 (2μM) prior to LPS stimulation. The cell extracts were analyzed by immunoblots with the indicated antibodies. The phospho-form of DCNL5 was visualized after immunoprecipitation as described in (B). **(E)** Same as (D), except that RAW264.7 were either mock treated or treated with MLN4924 (3μM) for 3 hours or MRT67307 (1μM) for 1 hour prior to LPS stimulation.

We next asked whether we could detect this phosphorylation event in cells, by generating a phospho-specific antibody raised against the Serine 41 epitope of DCNL5. Using this antibody we confirmed the phosphorylation of DCNL5 on Ser41 could be triggered by co-expression with IKKα in HEK293 cells (Fig 2A). Interestingly IKKα can also phosphorylate a DAD patch mutant of DCNL5, which no longer binds to Cullins, suggesting that this phosphorylation does not require Cullin interaction (S1 Fig).

Our phospho Ser41 antibody efficiently immunoprecipitated the endogenous phosphorylated form of DCNL5. Using this approach, we detected a clear induction of pSer41-DCNL5 10 minutes after TLR stimulation with lipopolysaccharide (LPS) in RAW264.7 macrophages, which gradually decreased over time (Fig 2B). LPS is a component of the outer membrane of gram-negative bacteria, it is recognized by the TLR4 receptor and stimulates both Myd88 and TRIF pathways [22]. Strikingly, the appearance of phosphorylated DCNL5 co-incided with Iκbα degradation and phosphorylation of p105, hallmarks of NF-Kb pathway activation (Fig 2B).

The stimulation of RAW 264.7 cells with other TLR agonists, such as Pam3CSK4 and Poly (I:C), also induced S41 phosphorylation of DCNL5 (Figs 1B and 2C). We further detected DCNL5 phosphorylation in MEFs and HEK293 cells stimulated with Interleukin-1 (IL-1), indicating that the phosphorylation of DCNL5 is not restricted to one specific cell type (S2 Fig).

We next used pharmacological inhibitors to confirm that IKKα phosphorylates DCNL5 specifically. We treated cells with MRT67307 (to inhibit TBK1/ IKKε), BI605906 (to inhibit IKKβ) or NG25 (to inhibit TAK1: the upstream kinase that activates both IKKα and IKKβ). While MRT67307 or BI605906 treatments did not affect DCNL5 phosphorylation, NG25 abolished it (Fig 2D). As NG25 inhibits both IKKα and IKKβ, while BI605906 only inhibits IKKβ, this result further strongly indicates that IKKα is the responsible kinase for DCNL5 phosphorylation in cells.

To determine if this phosphorylation also occurs in a physiologically relevant tissue, we immunoprecipitated DCNL5 from mouse spleen and thymus and found that it is phosphorylated in thymus (S3 Fig). The fact that we detected this phosphorylation within a tissue demonstrates that this modification exists *in vivo* and suggests that it is important for the biology of the organ itself.

### Activation of TLR signaling pathways does not induce a modification of Cullin neddylation levels

As the only known role of DCNLs is their function in Cullin neddylation [9,10], we sought to test whether the inducible phosphorylation of DCNL5 would alter the global Cullin neddylation profile. We first determined Cullin neddylation levels in RAW264.7 cells after treatment with LPS, but found that TLR stimulation does not induce any change in overall neddylation (Fig 2E). A pretreatment with MRT67307, which prevents phosphorylation of IRF3 and production of IFNβ, enhances the phosphorylation of DCNL5, but this also did not alter Cullin neddylation (Fig 2E).

Our previous immunofluorescence studies demonstrated that DCNL5 mainly localizes to the nucleus and plays an important role in the DNA damage response [9]. Consistently, cellular fractionation of RAW264.7 cells confirmed that the protein is mostly nuclear (S4). Upon TLR stimulation, DCNL5 is phosphorylated within the nucleus (S4 Fig), which is in accordance with the fact that IKKα has a predicted nuclear localization signal [23] and can shuttle from the cytoplasm to the nucleus [24]. Neddylation of Cullin1 was detectable in the nuclear fractions suggesting that active Cullin1 mainly exist in the nucleus. Subsequent analyses of the different Cullins revealed that all neddylated Cullins are predominantly located in the nucleus and that neddylated Cullin4a is predominantly present in chromatin-enriched fractions (S5 Fig) [25]. The NEDD8 E2 Ubc12 is also largely nuclear and nuclear localization of DCNL1 is important for its role as a Nedd8 E3 [26,27]. However, the neddylation status of the nuclear Cullin fraction remained unchanged after TLR stimulation (S5 Fig) and the nuclear localization of either DCNL5 or other Cullins was also unaffected (S5 Fig).

### DCNL5 Nedd8 E3 ligase activity is not changed upon Ser41 phosphorylation

**Fig 3:**
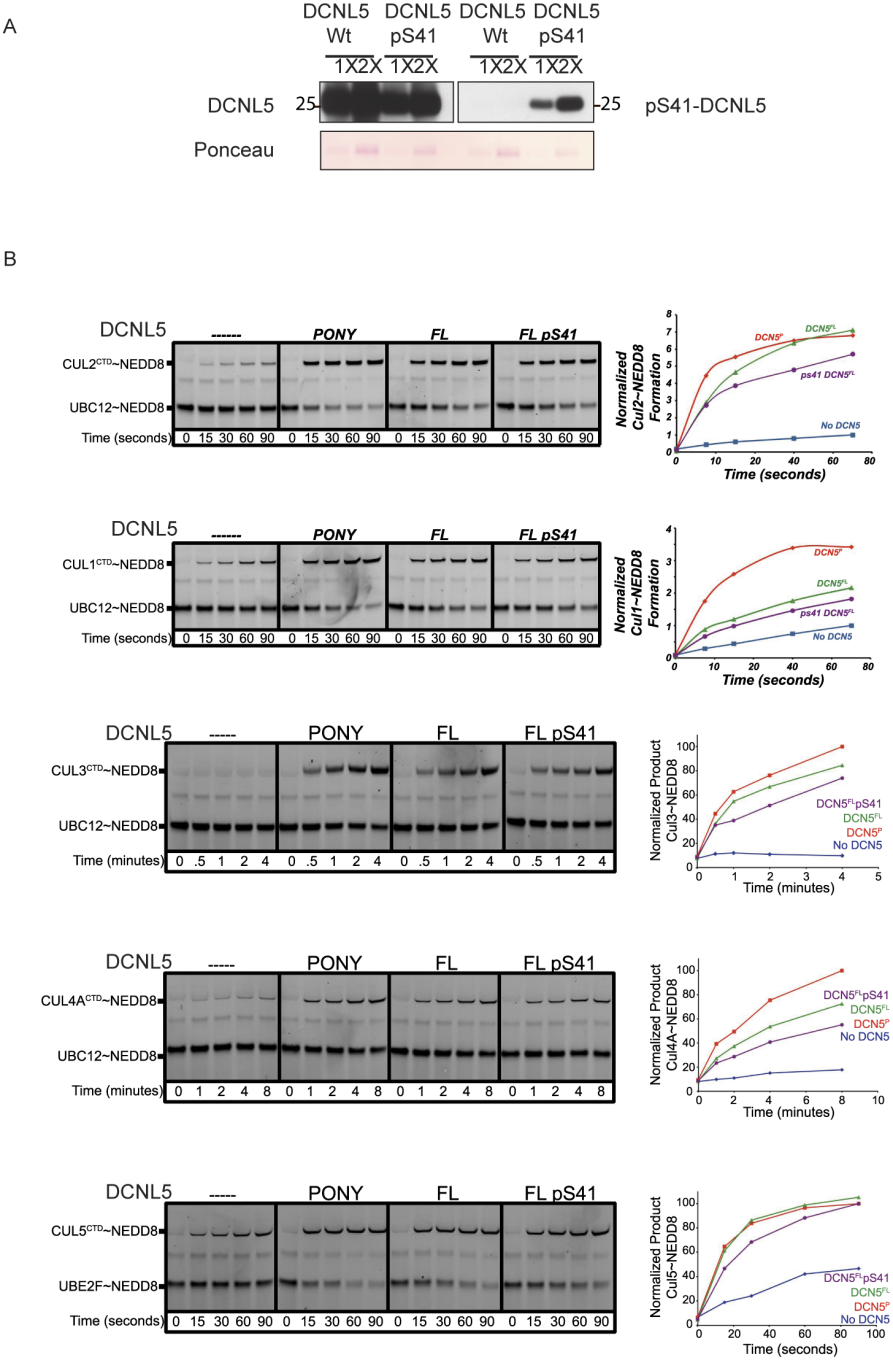
The phospho Serine 41 form of DCNL5 does not affect the kinetics of Cullin neddylation reactions. (A) EcAR7.SEP bacterial strain was transformed with plasmids harboring GST-DCNL5-WT or GST-DCNL5-S41TAG (the S41 codon was replaced by an amber stop codon). Purified proteins were cleaved from the GST tag using precision protease (GE) an analyzed by immunoblot with antibodies against DCNL5 or only the phospho form. **(B)** Pulse-chase [32P] NEDD8 transfer from UBC12NAc or UBE2FNAc (for Cullin 5 only) to the indicated Cullin C-terminal domain- RBX complexes in the absence or presence of the indicated DCNL5 constructs (PONY domain/ Full length and phosphorylated form. For comparison, all reactions were carried out under the same conditions.

To assess whether phosphorylation of DCNL5 has an effect on its Nedd8 E3 activity, we performed *in vitro* neddylation assays using recombinant DCNL5 and N-terminally truncated Cullins. To generate a recombinant DCNL5 phosphorylated on Ser41 we made use of an engineered system for specific co-translational O-phosphoserine (Sep) incorporation into the desired position of DCNL5 [14,28]. Although the incorporation was not 100% efficient we could clearly produce protein with significant incorporation of Ser41 as shown by mass spectrometry and western blot (Fig 3A and S6 Fig).

We monitored DCNL5 activity in the presence of the different Cullin fragments, which contained the entire C-terminal domain that mediates interactions with Rbx1/2 and the Nedd8 modification lysine. Briefly, after E1-mediated “pulse” generation of a thioester-linked E2- [^32^P]-NEDD8 intermediate, a Cullin CTD-RBX complex was added, and radiolabeled NEDD8 was “chased” from the E2 to the Cullin [8,29] [30]. In the absence of DCNL5, NEDD8 transfer to the Cullins is inefficient (Fig 3B, left panel). The addition of DCNL5 PONY domain potently stimulates NEDD8 transfer from E2 to Cullin CTD (2^nd^ panel from left). The use of full length DCNL5 does not further stimulate this transfer (3^rd^ panel) for Cullin2, Cullin3 and Cullin5, but somewhat lessens neddylation of Cullin1 and Cullin4a (Fig 3B, 2^nd^ and 4^th^ panel). These results suggest that *in vitro* the PONY domain is by itself sufficient for Cullin neddylation and that for some Cullins, the amino-terminus of the DCNL may attenuate neddylation, as was previously shown for DCNL3 [31]. The analyses of phosphorylated DCNL5 reveals similar transfer rates to the WT version, suggesting that the phosphorylation does not have a direct effect on Cullin neddylation and thus Nedd8 E3 ligase activity *in vitro* (4^th^ panel). However, we cannot exclude the possibility that in the more complex in vivo situation, phosphorylation might influence the activity in other ways.

### IKK mediated signaling pathways are not impaired in the absence of DCNL5

**Fig 4:**
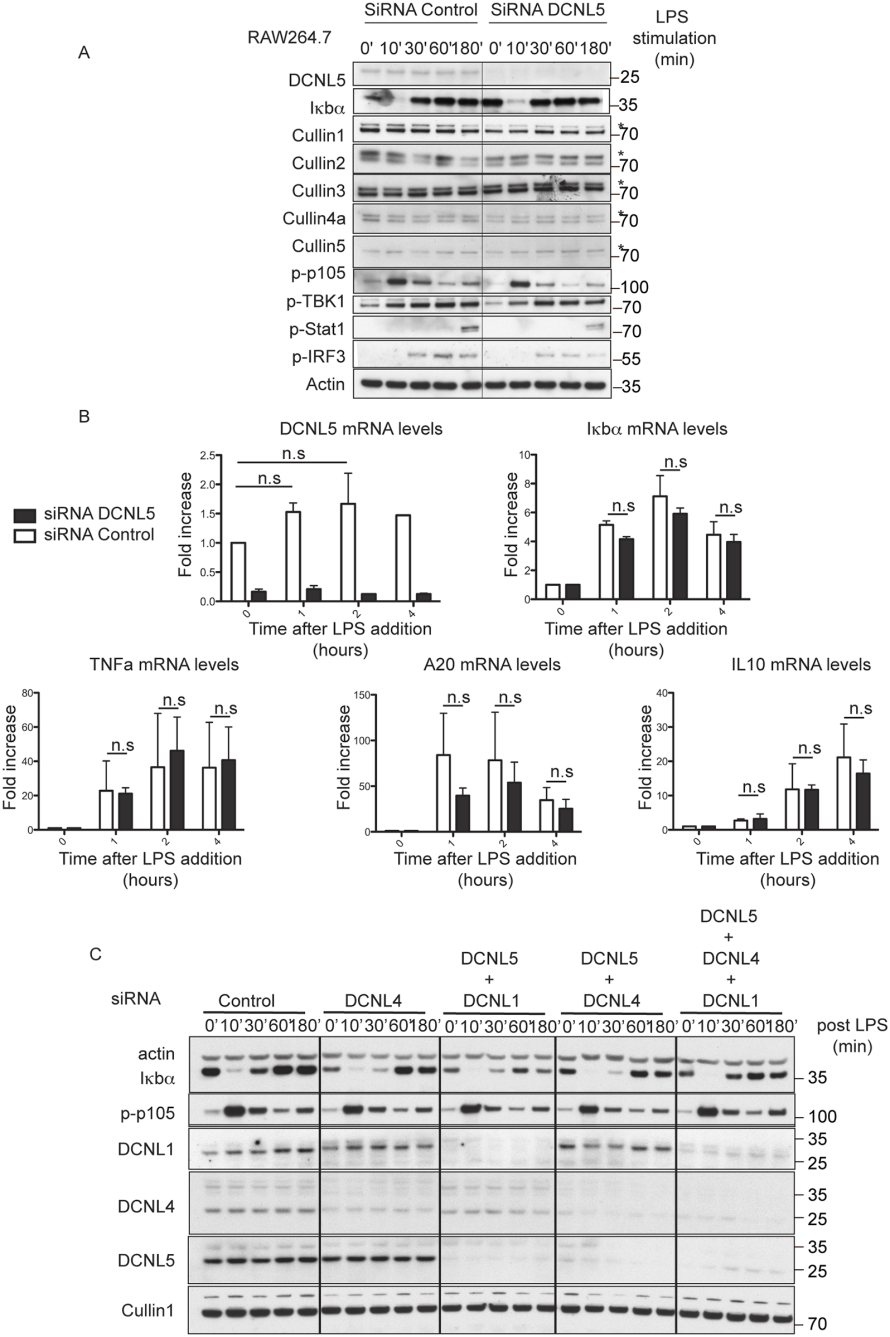
Knock down of DCNL5 by siRNA has no effect on the activation of the NF-KB pathway. (A) RAW264.7 were electroporated with siRNA and stimulated with LPS 24 hours later. Knockdown efficiency was measured by immunoblot. (B) As in (A) RAW264.7 were electroporated with siRNA and stimulated with Poly (I:C) 24 hours later for the indicated times. Total RNA was extracted and the mRNA encoding DCNL5, IFNβ, CXCL10, ISG15 and MX1 were measured by qRT-PCR as described under the “Experimental Procedures.” The results show relative mRNA levels compared with the value of 1.0 measured in unstimulated control cells. The experiment was performed in quadruplicate for each condition. Similar results were obtained in two independent experiments. Adjacent graphs show the means (± s.e.m) of quantified mRNA levels. Statistical significance was determined by two-ways ANOVA. P≤0.05 (C) RAW264.7 were electroporated with siRNA and stimulated with LPS 24 hours later. Knockdown efficiency was measured by immunoblot.

The IKK complex is the master regulator of the NF-KB pathway by controlling the degradation of Iκbα. The canonical IKK complex itself comprises three components, namely the protein kinases IKKα (also known as IKK1) and IKKβ (also known as IKK2) and NF-KB essential modulator (NEMO; also known as IKKγ) [32]. Previous studies shed light on the importance of IKKα in the resolution of inflammation by playing an inhibitory role in the NF-KB pathway independently of the IKK complex. IKKα is reported to specifically phosphorylate proteins such as TAX1BP1 or PIAS1 to control the degree of NF-KB pathway activation. TAX1BP1 is a scaffold protein, which together with Itch, RNF11 and A20 acts as a negative regulator of TRAF6 and RIP1 [33–37]. PIAS1 is a transcription factor with SUMO E3 ligase activity, the protein blocks binding of NF-KB transcription subunits to gene promoters [38,39]. The activity of TAX1BP1 and PIAS1 are positively controlled by IKKα phosphorylation. As IKKα specifically phosphorylates DCNL5, we sought to assess the contribution of DCNL5 in the regulation of the NF-KB pathway. For this we stimulated RAW264.7 cells with LPS after a DCNL5 knockdown by RNAi and followed the kinetics of degradation and re-synthesis of Iκbα as a read-out (Fig 4A). However, knock-down of DCNL5 neither affected the degradation nor the re-synthesis of Iκbα, indicating that DCNL5 is not involved in the control of Iκbα protein levels. Furthermore, DCNL5 knockdown does not impair phosphorylation of IRF3, a substrate of TBK1/IKKε, involved in type 1 IFN production. To confirm these results we also studied by qPCR mRNA production of IFNβ and IFNβ-dependent genes such as Isg15, CXCL10 (an established marker of viral infection) or MX1 [40,41] (S7 Fig). The knockdown of DCNL5 does not affect mRNA production of pro-inflammatory cytokines like TNFα or anti-inflammatory cytokines like IL-10 and also does not affect Iκbα or A20 mRNA synthesis, which are key inhibitors of the pathway critical for the resolution of inflammation [36,42] (Fig 4B). Altogether these results indicate that DCNL5 does not participate in the control of the NF-KB pathway. However, we have to consider the fact that in addition to DCNL5, the immune tissues analyzed also express DCNL1 and DCNL4 [9] and a phenotype may only be revealed in the absence of all DCNLs. RAW264.7 macrophages are immortalized cells, and so we cannot exclude the possibility that DCNL5, DCNL1 and DCNL4 function redundantly with one another, as has been observed in cancer cells [9].

Although DCNL5 is rapidly phosphorylated during TLR signaling and this correlates with Iκbα degradation, we were not able to find evidence of its involvement in Iκbα degradation or resynthesis. The fact that neither DCNL1 nor DCNL4 knock-down affect Iκbα degradation is puzzling (Fig 4C and S8 Fig): the SCF^βtrcp^ CRL complex degrades Iκbα and, in order to be active, CRLs need to be neddylated. We cannot exclude in this case that neddylation is not essential for full CRL activation. However, it has been shown previously that neddylation is crucial for LPS-induced cytokine production in macrophages. Blocking neddylation, either pharmacologically (MLN4924) or by using siRNA (against Nedd8 or Ubc12) abrogates the LPS-stimulated increase in certain pro-inflammatory cytokines secreted from macrophages in response to LPS (IL-6 and TNFα) [43], presumably by preventing the ubiquitylation and degradation of Iκbα.

## Discussion

DCNLs are facilitators of Cullin neddylation and thus important activators of the most abundant family of ubiquitin E3 ligases. Mutation or disregulation of Cullin–RING E3 ubiquitin ligases (CRLs) can result in the development of cancer and other human diseases. Targeting of CRL activation for pharmacological intervention may be a promising way to treat cancer. MLN4924, a small-molecule inhibitor of NAE that prevents neddylation and, therefore, activation of CRLs is in clinical trials for the treatment of hematological cancers [44]. The identification of specific regulation of Cullins by DCNLs in physiological contexts could thus facilitate the design and development of more targeted small molecule therapeutics.

In vitro, the five mammalian DCNLs don’t exhibit a particular Cullin preference, [8], which suggests that they could act redundantly in cells. However, while all DCNLs show relatively high expression level in cultured cancer cell lines, their expression is more restricted in animal tissues [9]. Furthermore, they all show unique subcellular distribution patterns, governed by their N-termini, indicating that their tissue specific expression and/or subcellular localization may mediate specificity [10,45]. DCNL5 is mostly nuclear and expressed in immune tissue [9]. From these observations we hypothesized that the protein could be involved in immune signaling. Consistent with this hypothesis, we identified DCNL5 as a substrate of IKKα after TLR activation. From this we initially hypothesized that DCNL5 may be activated during immune signaling to mediate the neddylation of Cullins. Of particular interest in this case would be the SCF^βtrcp^ Cullin complex, which is important for the degradation of Iκbα during TLR signaling. However, we did not detect any changes in SCF neddylation or Iκbα degradation in the absence of DCNL5 or during TLR signaling. We furthermore saw no differences in the neddylation state of any Cullins or any effect on the in vitro neddylation reactions in the presence of phosphorylated DCNL5. It is thus possible that DCNL5 phosphorylation is not directly involved in regulating neddylation. However, we may not be able to detect differences if only a subset of Cullin complexes are regulated. Cullins form potentially hundreds of different complexes with unique substrate adaptors and only a fraction of these may be affected by DCNL5 phosphorylation. Furthermore, it is possible that potentially specific effects are masked due to DCNL redundancy or ectopic activity from overexpression in cultured cells. We have recently noticed that cultured cancer cell lines express an unusually high amount of all DCNLs, and cultured immune cells may be similarly affected. To detect any specific phenotypes, it may be necessary to generate knockout and phosphorylation site knock-in mice that maintain tissue specific DCNL expression. In this regard, it would be hugely helpful to determine if DCNL5 mutant animals that are no longer able to phosphorylate Serine 41 display any immune defects.

It is also possible that the phosphorylation of DCNL5 is involved in yet unknown functions of DCNLs that are independent of Cullin neddylation, but directly impinge on other aspects of immunity. Given the highly specific phosphorylation of DCNL5 by IKKα and not IKKβ it is likely that IKKα dependent processes may be affected. In addition to its pro-inflammatory functions, IKKα is important for the resolution of inflammation. IKKα has been shown to phosphorylate TAX1BP1 [37] and PIAS1, the inhibitor of activated transcription factor STAT1 to downregulate pro-inflammatory signaling [38]. However, we were not able to show any involvement of DCNL5 in NF-KB activation or inhibition (exemplified by Iκbα degradation or resynthesis). DCNL5 might instead impact on the transcription of specific genes, which we have not tested. IKKα also participates in interferon production and responses downstream by directly phosphorylating interferon regulatory factor 7 (IRF7) [46]. In our experiments siRNA-mediated knockdown of DCNL5, however, did not affect interferon production, excluding the possibility that it regulates this aspect of the pathway.

Other than its role in the NF-KB signaling pathway, IKKα has been reported to phosphorylate a growing list of substrates that are involved in a variety of biological functions including tumor suppression [47] [48] [49], immune functions [50], cell proliferation [51,52], chromatin remodeling and transcription regulation [24,53,54]. DCNL5 phosphorylation may be involved in any of these processes. However, this seems unlikely, as the phosphorylation of DCNL5 is transient and closely tracks that of other immune regulators, such as p105.

IKKα is also a central component of the non-canonical NF-KB pathway. The kinase acts alone as a downstream effector of the central activating kinase of this pathway, the kinase NIK (NF-KB-inducing kinase). Compared to the canonical NF-KB pathway, activation of the non-canonical pathway involves different signaling molecules and is based on processing of the NF-KB2 precursor protein, p100 [55,56]. In MEFs and B cells, in response to developmental signals, such as lymphotoxin (LTa_1_b_2_), B-cell activating factor (BAFF) and CD40 ligand, IKKα induces p100 phosphorylation, thereby triggering p100 polyubiquitination by SCF^btrcp^ and proteasomal processing into p52 [57]. Hence DCNL5 phosphorylation could have a role in the non-canonical NF-KB pathway, but this needs to be tested more rigorously in the future.

In a more general context, and together with previous findings, our results further support the notion that the different amino-terminal domains of DCNL family members are important for their specificities. These amino-terminal domains may regulate activity through post-translational modifications such as myristoylation, ubiquitylation, phosphorylation or sumoylation. In that regard, the amino-terminal domain of DCNL5, which comprises the sequence surrounding the Serine 41, shows a high degree of conservation through evolution suggesting a potential important function (S9 Fig). The fact that we have not been able to identify a functional requirement for S41 phosphorylation of DCNL5 does not exclude this possibility, as to fully be able to define a role for DCNL5 in immune signaling, the generation of a transgenic animal model is paramount. Based on our ability to generate knock out cell lines, we would not expect the deletion of DCNL5 to induce lethality, [9], however we could speculate that the animals present with defects in their immune tissue and a have different sensitivity to infection compared to WT counterparts. Even in the unlikely case that phosphorylation of DCNL5 serves no biological function, it can serve as a useful biomarker for IKKα activity *in vivo*.

## Supporting information

S1 Fig Co-expression of DCNL5 DAD patch mutant with IKKα leads to phosphorylation on Serine 41 in HEK293 cells. Immunoblots of HEK293 cell lysates overexpressing Flag tagged DCNL5 WT, DAD (D195A, A219R) or S41A mutant and HA-tagged IKKα WT.
(TIF)

S2 Fig DCNL5 is phosphorylated on Serine 41 in HEK293 and MEFs upon stimulation with IL1b and IL1a respectively. **(A)** Same as Fig 2B and 2C using HEK293 stably expressing IL1 receptor and stimulated with human IL1**β** (5 ng/ml) **(B)** Same as (A) except MEFs were stimulated with mouse IL1**α** (5ng/ml).
(TIF)

S3 Fig DCNL5 phosphorylated on Serine 41 is detected in Spleen and Thymus mouse extracts. The phosphorylated form of DCNL5 was immunoprecipitated using the phospho-specific antibody from 3 mg of Spleen and Thymus mouse lysates and analyzed by immunoblot with a total DCNL5 antibody.
(TIF)

S4 Fig Upon TLR stimulation, DCNL5 is phosphorylated within the nucleus. (TIF)

S5 Fig The neddylation status of the nuclear Cullin fraction remained unchanged after TLR stimulation. RAW264.7 macrophages were treated with 100 ng/ml LPS and subjected to Cytoplasm and Nucleus fractionation followed by immunoblot analyses with the indicated antibodies. The phospho-form of DCNL5 was visualized after immunoprecipitation as described in Fig 2E.
(TIF)

S6 Fig Validation of phospho-serine insertion at S41 position. Top panel: Recombinant DCNL5 and DCNL5 pS41 were produced as described in Fig 3A. After SDS-PAGE electrophoresis, proteins were in gel digested with trypsin, alkylated and processed for MS analysis to verify the incorporation of phosphor-serine at position 41. Bottom panel: While the DCNL5 WT version did present any phosphorylated residues (panel 1, 3 and 5), the pS41 version exhibited specific phosphorylation at the position S41 (panel 2 and 4). However, strangely a small fraction of the pS41 DCNL5 protein contained Proline instead of Serine 41 (panel 6). NL: Intensity of the base peak; RT: Time range for averaging; m/z range 340–1800.
(TIF)

S7 Fig Knock down of DCNL5 by siRNA does not affect mRNA production of pro-inflammatory cytokines or anti-inflammatory cytokines. Same as in Fig 4B with the exception that the cells were stimulated with LPS for the indicated times. mRNA encoding DCNL5, Iκbα, A20, IL10 and TNFα were measured by qRT-PCR. The experiment was performed in quadruplicate for each condition. Similar results were obtained in three independent experiments. Adjacent graphs show the means (± s.e.m) of quantified mRNA levels. Statistical significance was determined by tow-ways ANOVA. P≤0.05.
(TIF)

S8 Fig The DCNL1 and DCNL5 knockdown have no effects on Iκbα degradation and resynthesis. RAW264.7 were electroporated with siRNA and stimulated with LPS 24 hours later. Knockdown efficiency was measured by immunoblot.
(TIF)

S9 Fig The amino terminal sequence of DCNL5 is well conserved through evolution. (TIF)
