# Supplementary figures and images for: The NEDD8 E3 ligase DCNL5 is phosphorylated by IKK alpha during Toll-like receptor activation
Source: PLoS One. 2018 Jun 29;13(6):e0199197. doi: 10.1371/journal.pone.0199197 (PMC6025869; doi:10.1371/journal.pone.0199197)

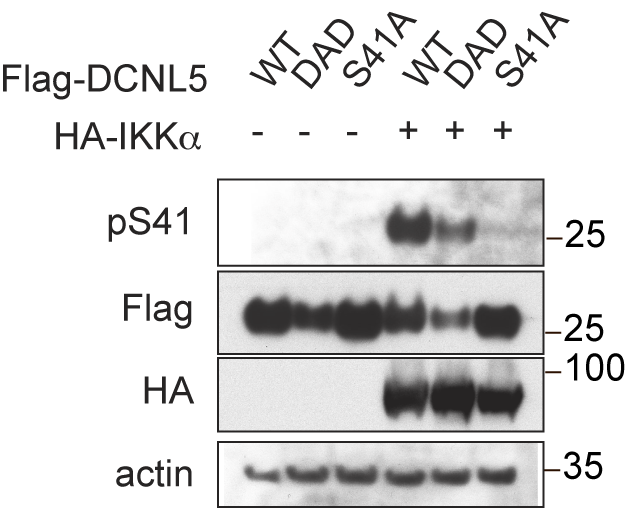

Supplement: S1 Fig — Immunoblots of HEK293 cell lysates overexpressing Flag tagged DCNL5 WT, DAD (D195A, A219R) or S41A mutant and HA-tagged IKKα WT. (TIF) [file pone.0199197.s001.tif]

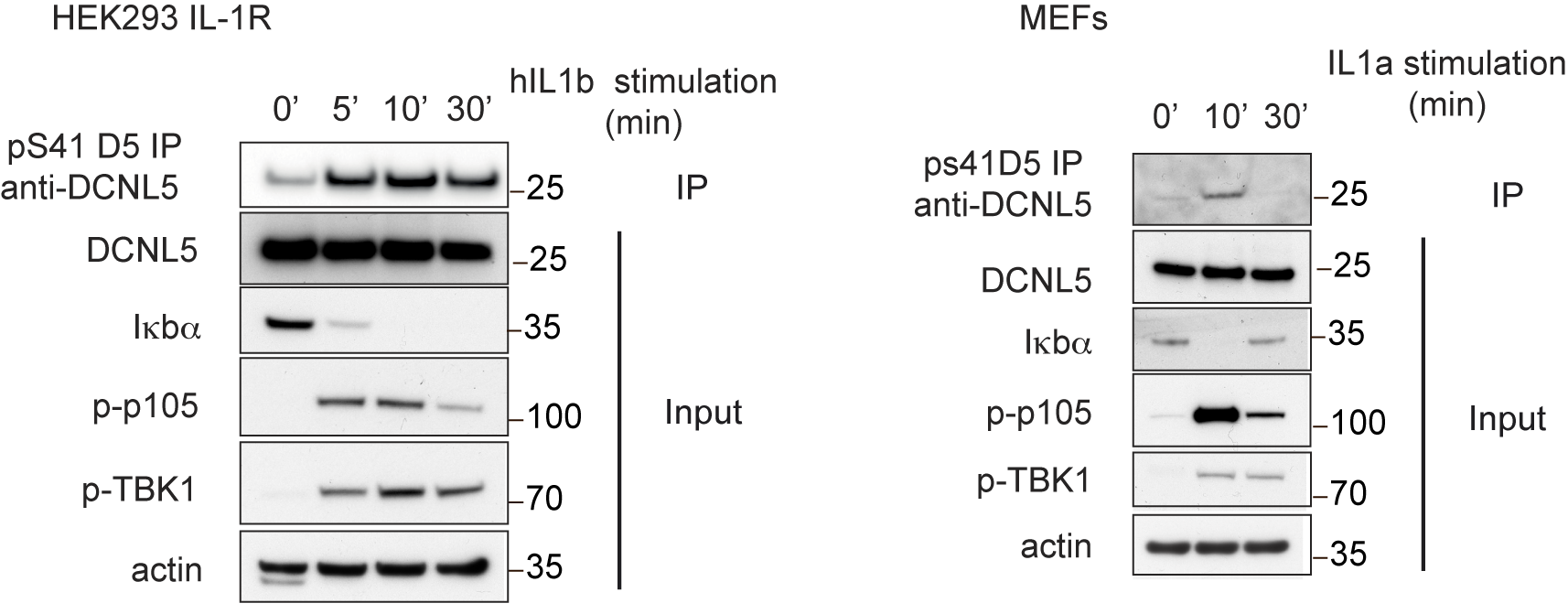

Supplement: S2 Fig — (A) Same as Fig 2B and 2C using HEK293 stably expressing IL1 receptor and stimulated with human IL1β (5 ng/ml) (B) Same as (A) except MEFs were stimulated with mouse IL1α (5ng/ml). (TIF) [file pone.0199197.s002.tif]

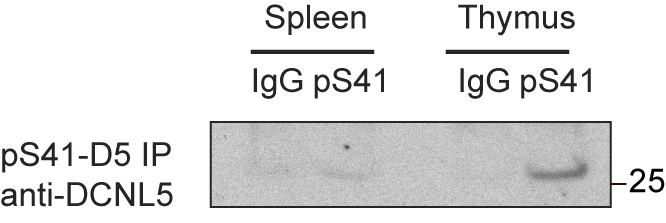

Supplement: S3 Fig — The phosphorylated form of DCNL5 was immunoprecipitated using the phospho-specific antibody from 3 mg of Spleen and Thymus mouse lysates and analyzed by immunoblot with a total DCNL5 antibody. (TIF) [file pone.0199197.s003.tif]

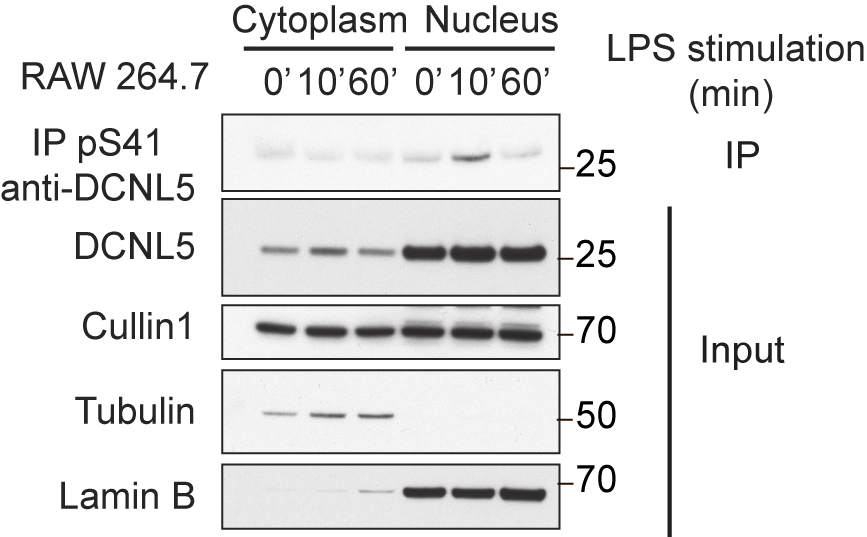

Supplement: S4 Fig — (TIF) [file pone.0199197.s004.tif]

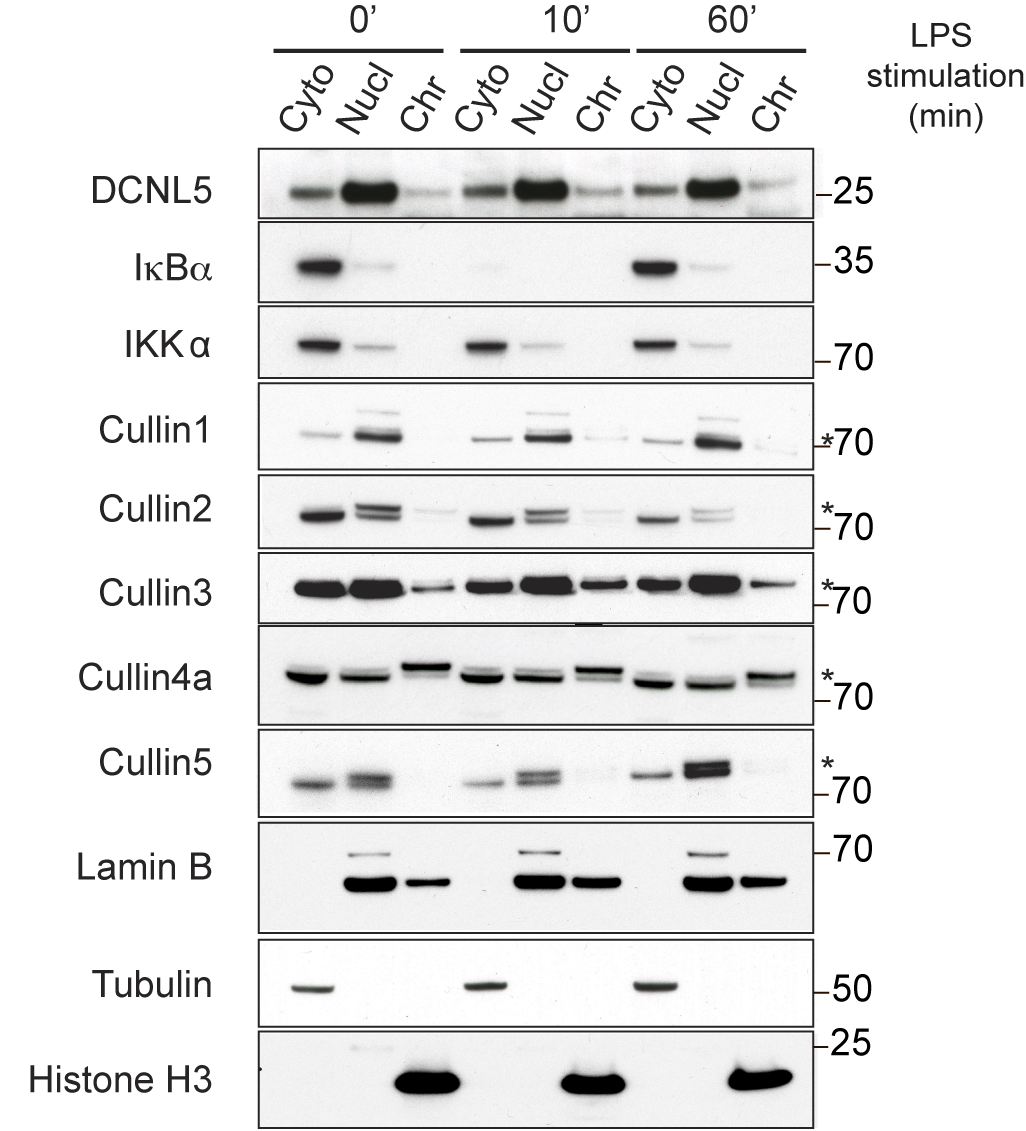

Supplement: S5 Fig — RAW264.7 macrophages were treated with 100 ng/ml LPS and subjected to Cytoplasm and Nucleus fractionation followed by immunoblot analyses with the indicated antibodies. The phospho-form of DCNL5 was visualized after immunoprecipitation as described in Fig 2E. (TIF) [file pone.0199197.s005.tif]

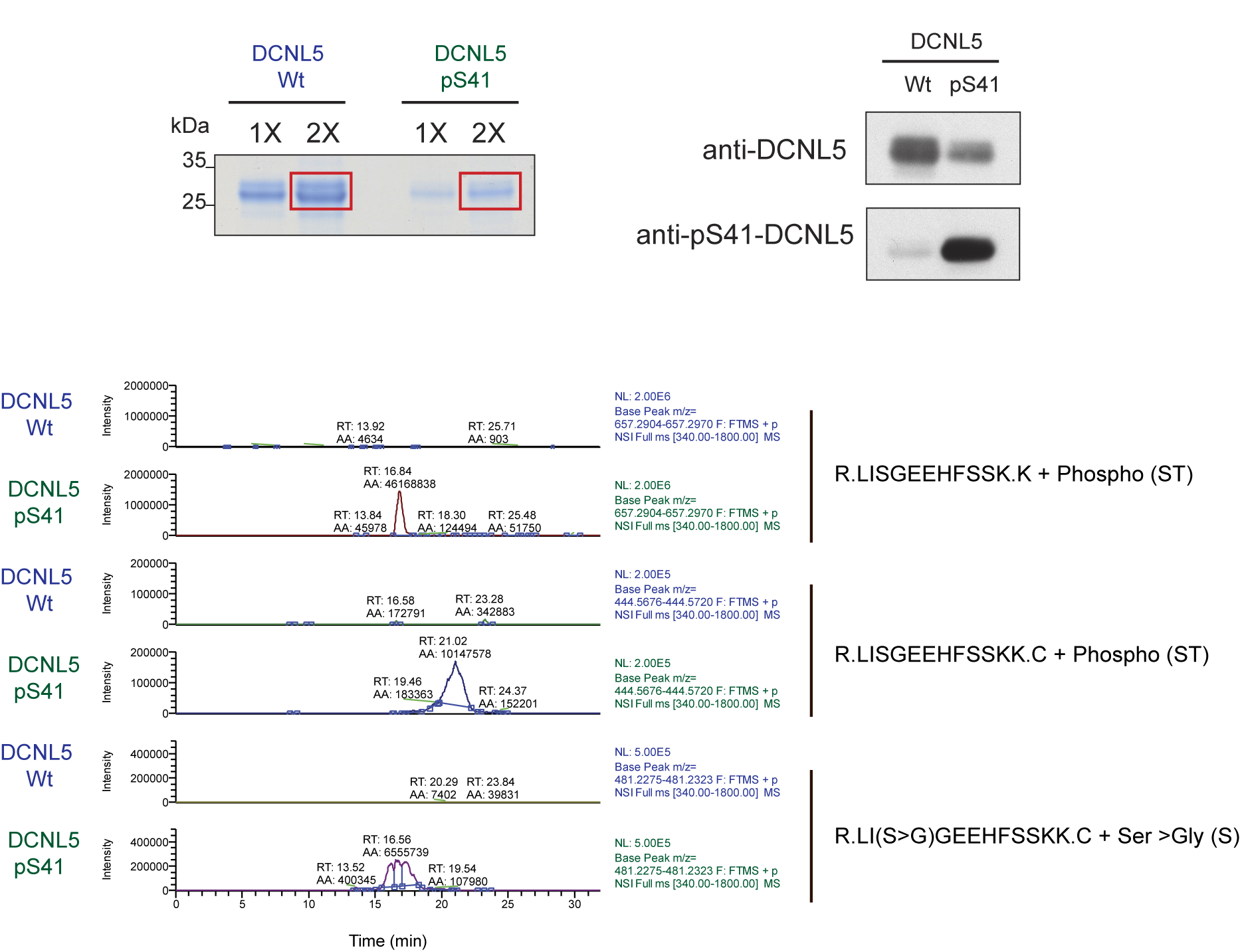

Supplement: S6 Fig — Top panel: Recombinant DCNL5 and DCNL5 pS41 were produced as described in Fig 3A. After SDS-PAGE electrophoresis, proteins were in gel digested with trypsin, alkylated and processed for MS analysis to verify the incorporation of phosphor-serine at position 41. Bottom panel: While the DCNL5 WT version did present any phosphorylated residues (panel 1, 3 and 5), the pS41 version exhibited specific phosphorylation at the position S41 (panel 2 and 4). However, strangely a small fraction of the pS41 DCNL5 protein contained Proline instead of Serine 41 (panel 6). NL: Intensity of the base peak; RT: Time range for averaging; m/z range 340–1800. (TIF) [file pone.0199197.s006.tif]

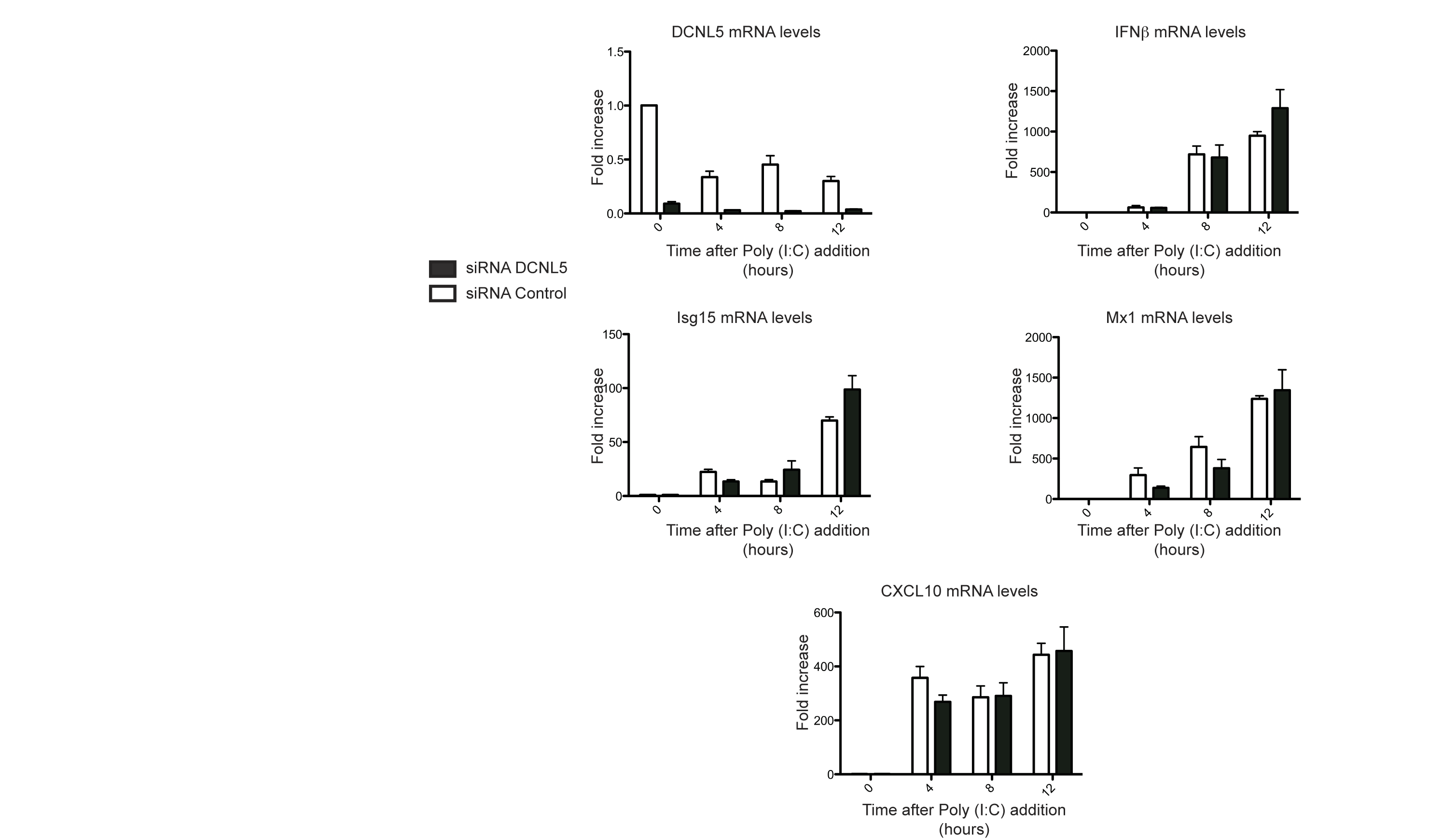

Supplement: S7 Fig — Same as in Fig 4B with the exception that the cells were stimulated with LPS for the indicated times. mRNA encoding DCNL5, Iκbα, A20, IL10 and TNFα were measured by qRT-PCR. The experiment was performed in quadruplicate for each condition. Similar results were obtained in three independent experiments. Adjacent graphs show the means (± s.e.m) of quantified mRNA levels. Statistical significance was determined by tow-ways ANOVA. P≤0.05. (TIF) [file pone.0199197.s007.tif]

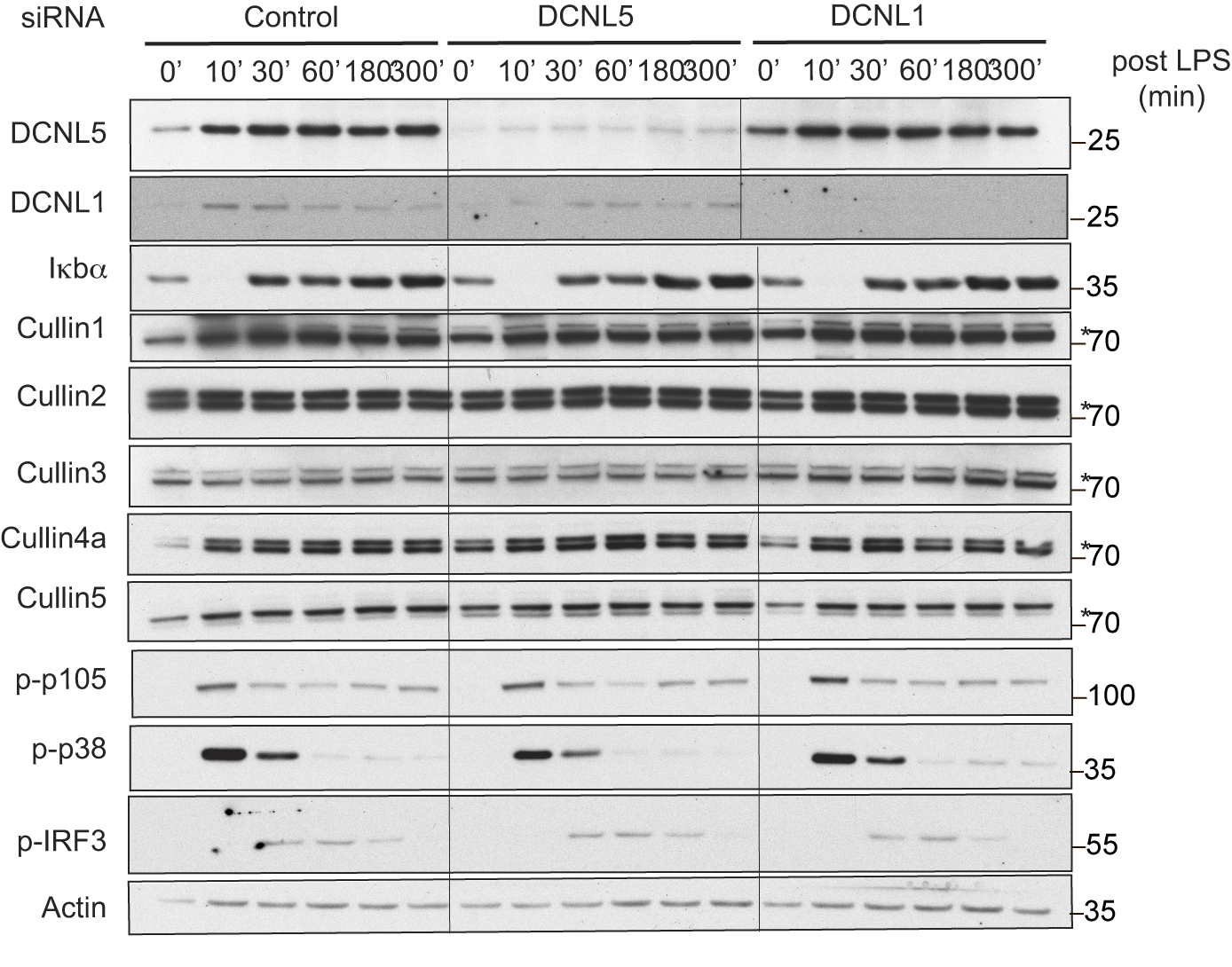

Supplement: S8 Fig — RAW264.7 were electroporated with siRNA and stimulated with LPS 24 hours later. Knockdown efficiency was measured by immunoblot. (TIF) [file pone.0199197.s008.tif]

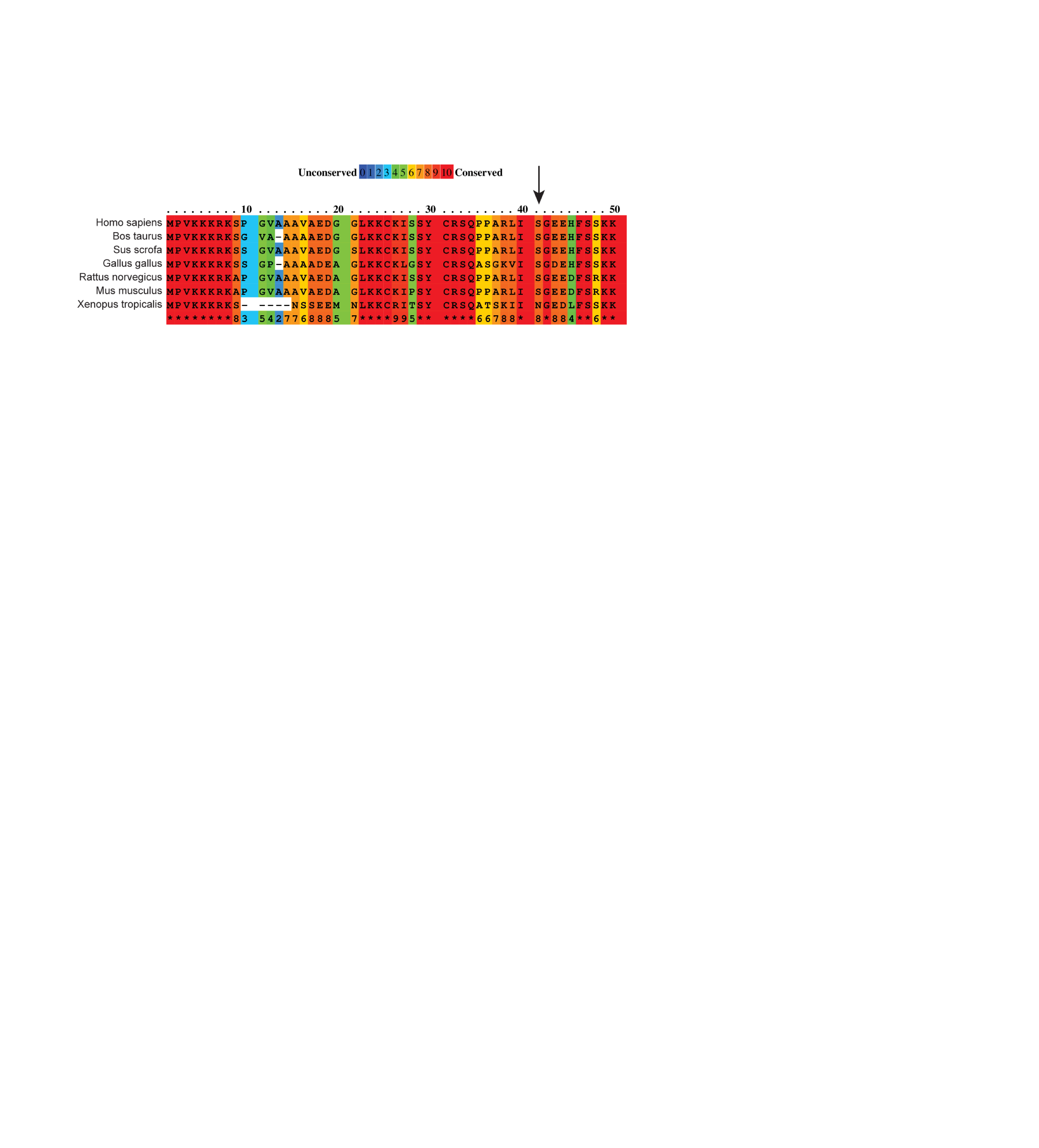

Supplement: S9 Fig — (TIF) [file pone.0199197.s009.tif]
